# Photobiomodulation Therapy Enhances Peripheral Nerve Repair by Restoring Nerve Growth Factor Levels and Increasing Myelin Protein Zero Expression After Chronic Constriction Injury in Rats

**DOI:** 10.3390/cimb48080806

**Published:** 2026-08-10

**Authors:** Mara Evany Oliveira, Fabio Martinez Santos, Adriano Polican Ciena, Marucia Chacur

**Affiliations:** 1 Functional Neuroanatomy of Pain Laboratory, Department of Anatomy, Institute of Biomedical Sciences, University of São Paulo (USP), São Paulo 05508-900, Brazil; maraevany@hotmail.com (M.E.O.); fabio.martinez@unifesp.br (F.M.S.); 2 Department of Morphology and Genetics, Paulista School of Medicine, Federal University of São Paulo (UNIFESP), São Paulo 04023-900, Brazil; 3 Morphology and Physical Activity Laboratory (LAMAF), Institute of Biosciences, São Paulo State University (UNESP), Rio Claro 13506-900, Brazil; adriano.ciena@unesp.br

**Keywords:** nerve injury, photobiomodulation, sciatic nerve, neural regeneration, peripheral neuropathy

## Abstract

Neuropathic pain is a chronic condition frequently associated with peripheral nerve injury and is characterized by significant structural and molecular alterations. Photobiomodulation therapy (PBMT) has demonstrated analgesic and anti-inflammatory effects; however, its influence on neurotrophic and myelin-associated repair mechanisms remains incompletely understood. The aim of this study was to investigate the effects of PBMT on ultrastructural, morphometric, and molecular changes induced by chronic constriction injury (CCI) of the sciatic nerve in male rats, with an emphasis on NGF and P0 expression. Neuropathy was induced using the CCI model of the sciatic nerve. PBMT was initiated 14 days after injury and administered in ten sessions using an infrared 904 nm laser. Transmission electron microscopy, morphometric analysis, immunofluorescence, and Western blot analyses were performed to evaluate neural regeneration and the expression of NGF and P0. Animals subjected to CCI exhibited ultrastructural changes consistent with Wallerian degeneration, accompanied by reduced NGF and P0 expression in the sciatic nerve. In contrast, PBMT markedly preserved nerve morphology, restored NGF expression, and significantly increased P0 protein levels. Together, these findings demonstrate that PBMT enhances peripheral nerve repair by promoting both molecular and structural mechanisms involved in axonal regeneration and myelin restoration. Restoration of NGF expression and increased P0 protein expression suggest that these mechanisms contribute to the regenerative effects of PBMT, supporting its potential as a safe, noninvasive therapeutic strategy for peripheral neuropathies.

## 1. Introduction

Neuropathic pain is a chronic condition resulting from injury or disease affecting the somatosensory system and is frequently characterized by spontaneous pain, hyperalgesia, and allodynia. It is estimated that approximately 7% of the global population experiences some degree of neuropathic pain, a condition associated with substantial functional impairment, reduced quality of life, and significant socioeconomic burden. The mechanisms underlying its pathophysiology include peripheral and central sensitization, alterations in neuronal excitability, and activation of neuroinflammatory pathways that contribute to the maintenance of the painful state [1,2,3,4].

Experimental models of peripheral nerve injury have been widely used to investigate the cellular and molecular mechanisms associated with neuropathic pain. Among these models, chronic constriction injury (CCI) of the sciatic nerve, originally described by Bennett and Xie, reproduces several features observed in human neuropathies, including mechanical and thermal hyperalgesia, allodynia, and progressive structural alterations in the injured nerve. Following injury, Wallerian degeneration occurs and is characterized by axonal fragmentation, degradation of the myelin sheath, macrophage recruitment, and extensive reorganization of the neural microenvironment, all of which are fundamental events for the subsequent regenerative process [5,6,7].

Regeneration of the peripheral nervous system depends on coordinated interactions among neurons, Schwann cells, and neurotrophic factors. Among these mediators, nerve growth factor (NGF) plays a central role in neuronal survival, maintenance of the sensory neuronal phenotype, and axonal regeneration following injury. Alterations in NGF expression have been associated with both the development of neuropathic pain and the functional recovery capacity of peripheral nerves. Previous studies have demonstrated that modulation of this factor directly influences axonal growth, neural plasticity, and tissue reorganization following nerve injury [8,9,10,11].

Another essential component of structural recovery following peripheral nerve injury is myelin protein zero (P0), the major structural protein of peripheral nervous system myelin. Produced by Schwann cells, P0 participates in the compaction and stabilization of the myelin sheath and is considered an important marker of myelin repair. Alterations in the expression of this protein have been associated with myelination defects and impaired nerve conduction, whereas its restoration has been linked to peripheral nerve repair and functional recovery following nerve injury [12,13]. Over the past years, photobiomodulation therapy (PBMT), also known as low-level laser therapy, has attracted increasing attention because of its anti-inflammatory, analgesic, and regenerative effects. Experimental studies have demonstrated that this therapeutic modality is capable of reducing mechanical and thermal hypersensitivity, modulating glial activation, decreasing pro-inflammatory cytokine production, and attenuating the expression of nociceptive mediators associated with neuropathic pain. In addition, PBMT presents favorable clinical characteristics, including low cost, noninvasive application, and a low incidence of adverse effects [14,15,16].

More recently, PBMT has been shown to reduce the expression of substance P and TRPV1 in dorsal root ganglia, as well as normalize thermal alterations associated with neuropathic pain induced by sciatic nerve injury. These findings further support the potential of photobiomodulation as a therapeutic strategy for nociceptive modulation. However, despite the evidence regarding pain control, it remains unclear whether the benefits of PBMT extend to mechanisms directly involved in peripheral nerve repair, particularly those related to the expression of neurotrophic factors and proteins associated with myelin repair [17].

Therefore, the hypothesis of the present study is that photobiomodulation not only reduces neuropathic pain induced by chronic constriction injury of the sciatic nerve but also promotes a biological environment favorable to peripheral nerve regeneration. Accordingly, the aim of this study was to investigate the effects of PBMT on ultrastructural, and molecular alterations associated with peripheral nerve injury, with particular emphasis on the expression of nerve growth factor (NGF) and myelin protein zero (P0), two key molecular markers involved in axonal regeneration and myelin repair.

To address this objective, transmission electron microscopy, morphometric analysis, immunofluorescence, and Western blot analyses were employed to evaluate the effects of PBMT on peripheral nerve repair following chronic sciatic nerve injury. By integrating ultrastructural and molecular approaches, this study provides a comprehensive characterization of the mechanisms underlying the therapeutic effects of photobiomodulation in experimental peripheral neuropathy.

## 2. Methods

### 2.1. Animals

Sixty male Wistar rats weighing 200–220 g were randomly assigned to four experimental groups (*n* = 15 animals per group). Animals were housed under controlled temperature and lighting conditions on a 12 h light/dark cycle, with free access to food and water throughout the experimental period. Animals were housed in polypropylene cages containing wood-shaving bedding under standard environmental conditions. Animals were obtained from the Central Animal Facility of the Institute of Biomedical Sciences, University of Sao Paulo, Brazil. All experimental procedures were approved by the Animal Care and Use Committee of the Institute of Biomedical Sciences, University of Sao Paulo (Protocols No. 091, Book 02/2012, and 2017110820) and were conducted in accordance with institutional guidelines and internationally accepted principles for the care and use of laboratory animals [18].

Animals were randomly assigned to four experimental groups: naïve—animals that did not undergo any surgical procedure; SHAM—sham-operated animals; CCI—animals subjected to chronic constriction injury of the sciatic nerve; and CCI + PBMT—animals subjected to chronic constriction injury of the sciatic nerve and treated with PBMT. The experimental unit was one animal. Sample size was based on previous studies from our laboratory using the same experimental model and similar outcome measures. No a priori sample size calculation was performed. Animals were randomly allocated to the experimental groups using a simple randomization procedure. No animals or experimental data were excluded from the analyses. All animals completed the experimental protocol and were included in the final statistical analyses.

### 2.2. Induction of Neuropathic Pain

Neuropathic pain was induced using the chronic constriction injury (CCI) model of the sciatic nerve, as originally described by [6]. Animals were anesthetized with isoflurane (Cristália, Sao Paulo, Brazil), and the right sciatic nerve was surgically exposed through an incision in the mid-thigh region. Proximal to the sciatic nerve trifurcation, four loose ligatures of 4-0 chromic catgut were placed around the nerve, approximately 1 mm apart, producing a mild constriction without completely interrupting nerve conduction. Animals in the SHAM group underwent the same surgical exposure of the sciatic nerve; however, no ligatures were applied. Following surgery, animals were monitored until complete recovery from anesthesia and were observed daily throughout the experimental period. Animals were monitored daily throughout the experimental period for signs of pain, distress, and postoperative complications.

### 2.3. Photobiomodulation Therapy

Treatment was initiated on postoperative day 14, corresponding to the established phase of experimental neuropathy. Photobiomodulation therapy (PBMT) was delivered using a LASERPULSE Diamond^®^ device (IBRAMED, São Paulo, Brazil) equipped with a gallium arsenide (GaAs) laser emitter. This treatment window was selected because postoperative day 14 corresponds to the established phase of neuropathic pain in the CCI model, when inflammatory and degenerative processes are already well established. The irradiation parameters (904 nm, 6 J/cm^2^) were selected based on previous experimental studies from our group demonstrating antinociceptive and neuroprotective effects using the same protocol, thereby allowing direct comparison with our previous findings [17,19]. Future studies should investigate whether earlier intervention or alternative irradiation parameters produce different regenerative outcomes. Treatments were performed on alternate days for a total of ten sessions. Laser irradiation was applied in contact mode to the skin overlying the course of the right sciatic nerve, with nine irradiation points distributed along the affected hind limb. The parameters used were: wavelength of 904 nm, frequency of 9500 Hz, beam area of 0.17 cm^2^, irradiation time of 18 s per point, and energy density of 6 J/cm^2^. Each session delivered a total radiant energy of 9.18J over a treated area of 1.53 cm^2^. The complete protocol resulted in a cumulative radiant energy of 91.9 J across the treatment period [19]. The irradiation parameters are summarized in Table 1.

### 2.4. Transmission Electron Microscopy

At the end of the experimental protocol, animals were anesthetized by intraperitoneal injection of urethane (3 g/kg) and transcardially perfused with a modified Karnovsky fixative solution containing 2.5% glutaraldehyde and 2% paraformaldehyde in 0.1 M sodium phosphate buffer (pH 7.4), as previously described by [8,20]. Following perfusion, the right sciatic nerve was carefully dissected, and fragments approximately 3 mm in length were collected for ultrastructural analysis. Samples were post-fixed in 1% osmium tetroxide at 4 °C and subsequently immersed in a 5% aqueous uranyl acetate solution at room temperature. Tissues were then dehydrated through a graded ethanol series, transferred to propylene oxide, and embedded in Spurr resin. Semi-thin sections were obtained using a Reichert Ultra Cut^®^ (Vienna, Austria) ultramicrotome and stained with 1% toluidine blue for identification of areas of interest. Subsequently, ultrathin sections approximately 60 nm thick were collected on 200-mesh copper grids (Sigma-Aldrich, St. Louis, MO, USA) and contrasted with 4% uranyl acetate and 0.4% lead citrate, according to the method described by [21]. Samples were examined using a JEOL JEM-1010 transmission electron microscope (JEOL Ltd., Tokyo, Japan) located in the Department of Anatomy, Institute of Biomedical Sciences, University of São Paulo. Qualitative analysis was performed to evaluate nerve fiber integrity, myelin sheath organization, the presence of myelinated and unmyelinated fibers, alterations consistent with Wallerian degeneration, and morphological features suggestive of neural regeneration following photobiomodulation therapy. In addition, morphometric analysis of the electron micrographs was performed to determine myelin sheath thickness, nerve fiber diameter, and axon diameter. The resulting measurements were used for comparisons among the experimental and control groups. Representative images were selected from fields that best reflected the overall ultrastructural characteristics consistently observed within each experimental group.

### 2.5. Morphometric Analysis

Transmission electron microscopy micrographs were used for morphometric analysis of sciatic nerve fibers. The following structural parameters were evaluated: myelin sheath thickness, nerve fiber diameter, and axon diameter. Measurements were performed on representative images obtained from the different experimental groups (NAIVE, SHAM, CCI, and CCI + PBMT) using ImageJ software (version 1.51r; National Institutes of Health, Bethesda, MD, USA). A total of 100 myelinated nerve fibers obtained from five animals per group were analyzed. Myelin sheath thickness was determined by measuring the myelin layer surrounding the axon. Nerve fiber diameter was defined as the total diameter of the myelinated fiber, whereas axon diameter corresponded to the direct measurement of the axon in cross section. Morphometric analyses were performed by a blinded investigator. Results were expressed in micrometers (µm) and presented as mean ± standard error of the mean (SEM). The obtained data were used to compare ultrastructural alterations induced by chronic constriction injury of the sciatic nerve and to evaluate the effects of photobiomodulation therapy on the preservation and regeneration of nerve fibers.

### 2.6. Immunofluorescence

Following induction of neuropathy and completion of the treatment protocol, animals were anesthetized with a ketamine/xylazine mixture (1:1; 100 μL/100 g body weight) and transcardially perfused with saline followed by 4% paraformaldehyde. The sciatic nerves were collected, post-fixed for 4 h, and subsequently transferred to a 30% sucrose solution for cryoprotection. After 24 h, tissues were sectioned at 14 μm thickness using a cryostat. Sections were incubated with primary antibodies against NGF and P0 (1:1000; Santa Cruz Biotechnology), followed by incubation with a TRITC-conjugated secondary antibody (1:100; Jackson ImmunoResearch, Chester County, PA, USA). Immunoreactivity was evaluated using a Nikon Eclipse 80i microscope, Tokyo, Japan equipped with an epifluorescence system and a Nikon DXM 1200F digital camera, Tokyo, Japan. Approximately fifteen sections per experimental group were qualitatively evaluated to assess the distribution patterns of NGF and P0 immunoreactivity. No semiquantitative fluorescence intensity analysis was performed, and immunofluorescence images were used as representative qualitative evidence supporting the ultrastructural and Western blot findings.

### 2.7. Western Blot Analysis

Following completion of the experimental protocols, fresh sciatic nerve tissue was rapidly collected, immediately snap-frozen in liquid nitrogen, and stored at −80 °C until protein extraction. Western blot analysis was performed as previously described by [22,23] with minor modifications. Total protein concentration was determined using the Bradford method [24]. Samples were homogenized in 125 mM Tris/HCl buffer (pH 6.8) containing 2.5% SDS, 2.5% 2-mercaptoethanol, 4 mM EDTA, and 0.05% bromophenol blue. Proteins were separated by SDS-polyacrylamide gel electrophoresis (10% SDS-PAGE) according to [25] and transferred onto nitrocellulose membranes (Millipore, Burlington, MA, USA) as described by [26]. Membranes were blocked with 5% nonfat dry milk and incubated overnight (18 h) at 4 °C with the following primary antibodies: anti-NGF (F30; 1:1000; Santa Cruz Biotechnology, Dallas, TX, USA); anti-P0 (1:1000; Abcam, Cambridge, UK). Subsequently, membranes were incubated with horseradish peroxidase-conjugated goat anti-rabbit IgG secondary antibody (1:5000; Chemicon, Tokyo, Japan). β-actin (1:10,000; Sigma, Kanagawa, Japan) was used as the endogenous loading control. Protein bands were visualized using an enhanced chemiluminescence detection system (ECL; Amersham Biosciences, Buckinghamshire, UK). Optical density of the immunoreactive bands was quantified using Scion Image software version Beta 4.0.2 (Scion Corporation, Chicago, IL, USA) and normalized to β-actin expression [22,23]. Western blot analyses were performed using sciatic nerve samples obtained from five independent animals per experimental group.

### 2.8. Statistical Analysis

Data are presented as mean ± standard error of the mean (SEM). Normality was assessed using the Shapiro–Wilk test, and homogeneity of variance was verified before performing parametric statistical analyses. Statistical analyses were performed using GraphPad Prism software 8.0 (GraphPad Software Inc., San Diego, CA, USA). Behavioral data were analyzed using two-way repeated-measures analysis of variance (ANOVA), followed by Bonferroni’s post hoc test for multiple comparisons. Morphometric and Western blot data were analyzed using one-way ANOVA followed by Bonferroni’s post hoc test. Differences were considered statistically significant when *p* < 0.05. Morphometric measurements, immunofluorescence analyses, transmission electron microscopy evaluation, and Western blot quantification were performed by investigators blinded to the experimental groups. Whenever applicable, quantitative analyses were performed by investigators blinded to experimental allocation.

## 3. Results

### 3.1. Ultrastructural Analysis of the Sciatic Nerve

**Figure 1:**
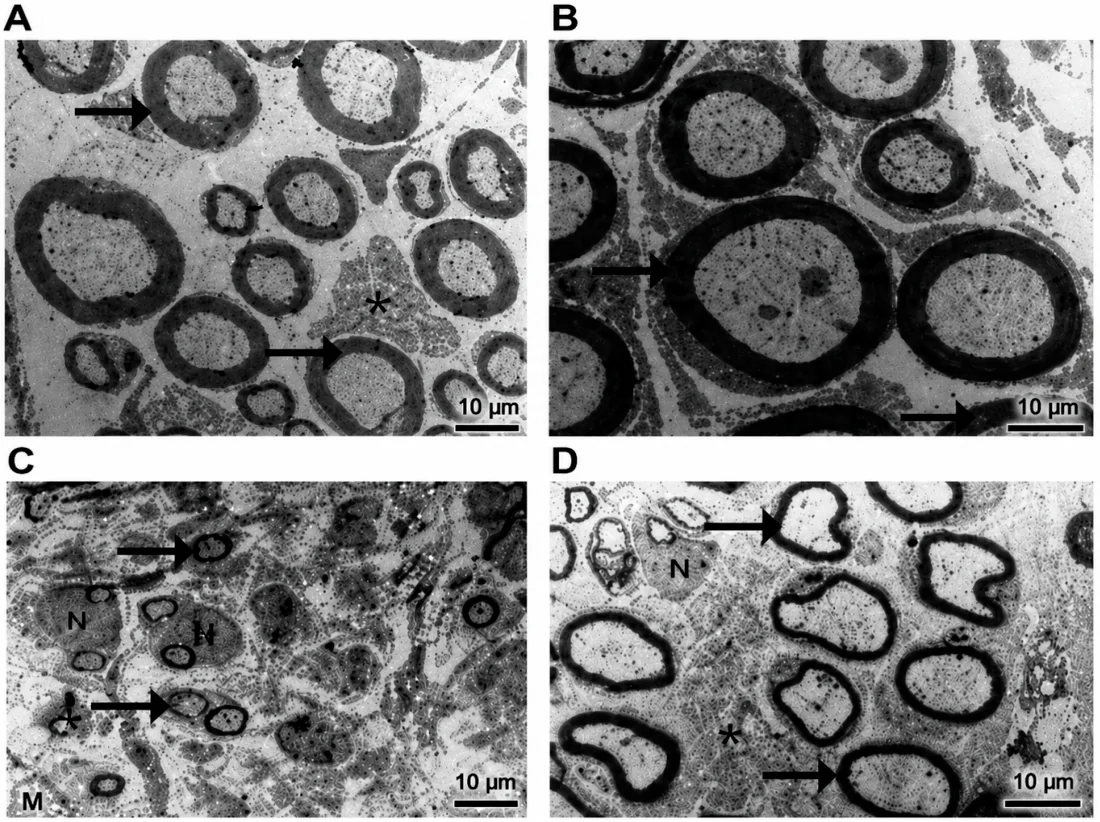
**Representative transmission electron micrographs of the right sciatic nerve.** (**A**) NAIVE and (**B**) SHAM groups exhibit preserved nerve fiber organization, with regularly distributed myelinated and unmyelinated fibers. (**C**) The CCI group shows alterations consistent with Wallerian degeneration, myelin disorganization, and reduced structural integrity. (**D**) The CCI + PBMT group exhibits myelinated fibers with features suggestive of neural regeneration and improved preservation of nerve architecture. Scale bar = 10 µm. Abbreviations: N, Schwann cell nucleus; M, macrophage; asterisks, unmyelinated fibers; arrows, myelinated fibers.

Ultrastructural analysis of the sciatic nerve revealed marked differences between the control and experimental groups. In the NAIVE and SHAM groups, the nerve architecture was preserved, characterized by the regular distribution of nerve fibers, homogeneous fascicular organization, and intact myelin sheaths. Myelinated fibers exhibited well-defined contours and compact myelin lamellae. In addition, unmyelinated fibers were uniformly distributed throughout the endoneurium, with no evident signs of structural degeneration. Schwann cell nuclei displayed preserved morphology consistent with normal peripheral nerve tissue (Figure 1).

In contrast, animals subjected to chronic constriction injury (CCI) exhibited ultrastructural alterations consistent with Wallerian degeneration. A reduction in nerve fiber density, disorganization of neural tissue, and the presence of degenerating myelinated fibers were observed. In several regions, the myelin sheath displayed loss of lamellar organization, irregular morphology, and structural disruption compatible with nerve injury-induced degeneration. Although some preserved myelinated fibers could still be identified, morphological features indicative of chronic neural damage predominated (Figure 1).

Animals treated with photobiomodulation therapy (CCI + PBMT) exhibited a distinct morphological pattern compared with untreated CCI animals. Transmission electron micrographs demonstrated greater preservation of nerve fiber structure and evidence of myelin repair. In several regions, myelinated fibers displayed features suggestive of remyelination, characterized by a more regular organization of myelin lamellae compared with the untreated CCI group. Moreover, the overall neural architecture appeared better preserved, suggesting a protective and regenerative effect of PBMT on the injured peripheral nerve.

Taken together, these ultrastructural findings demonstrate that chronic constriction injury induced degenerative changes consistent with peripheral neuropathy, whereas photobiomodulation therapy promoted preservation of the neural structure and supported the nerve repair process (Figure 1).

### 3.2. Morphometric Analysis of the Sciatic Nerve

**Figure 2:**
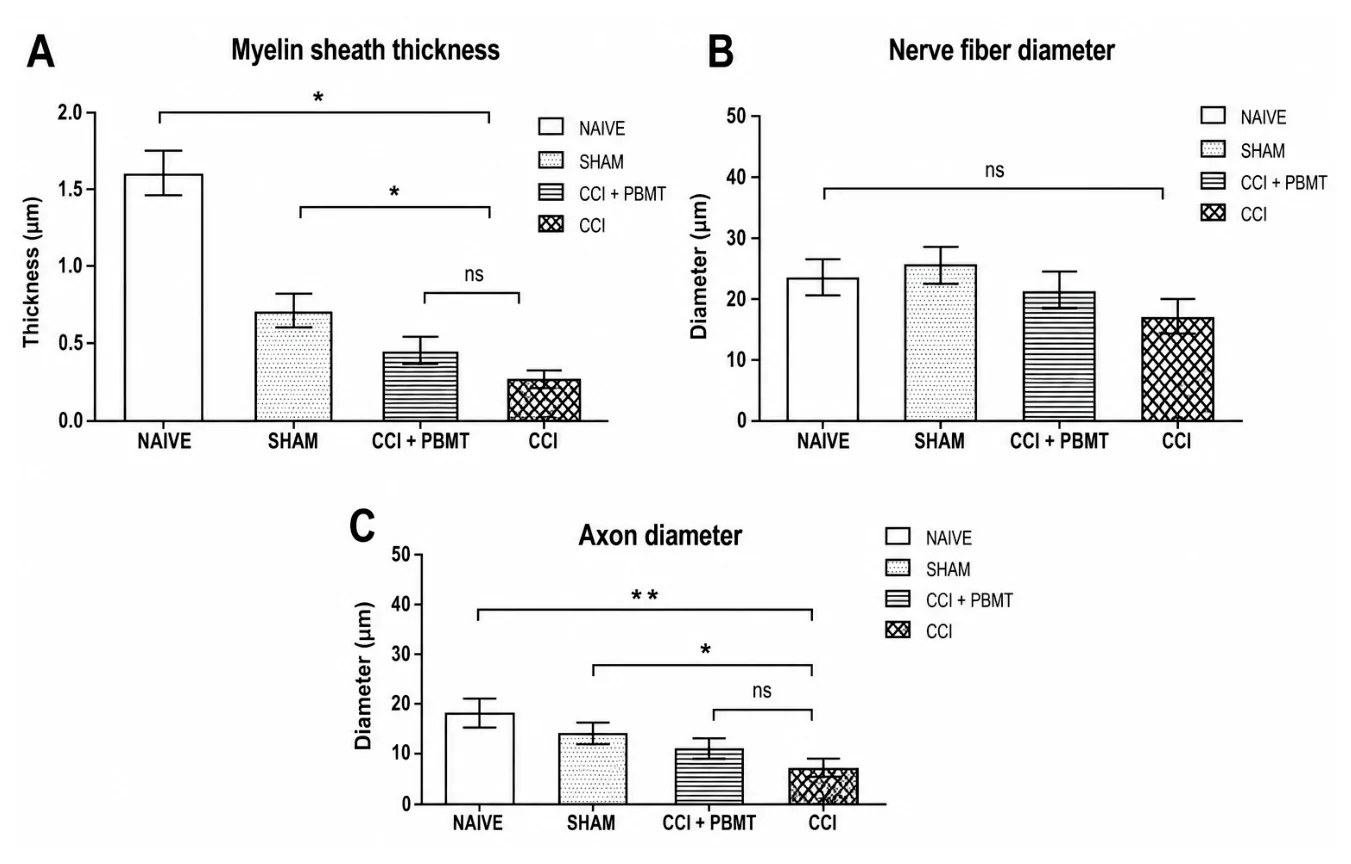
**Morphometric analysis of the sciatic nerve following chronic constriction injury (CCI) and photobiomodulation therapy (PBMT).** Morphometric evaluation of (**A**) myelin sheath thickness, (**B**) nerve fiber diameter, and (**C**) axon diameter in the NAIVE, SHAM, CCI, and CCI + PBMT groups. Data are presented as mean ± SEM. Statistical analysis was performed using one-way ANOVA followed by Tukey’s post hoc test, with comparisons conducted among all experimental groups. Only the pairwise comparisons relevant to the principal findings are displayed in the graphs. * *p* < 0.05; ** *p* < 0.01; ns, not significant.

Morphometric analysis was performed to quantitatively assess the structural alterations induced by chronic constriction injury (CCI) and the effects of photobiomodulation therapy (PBMT) on peripheral nerve morphology. CCI induced marked structural impairment in the sciatic nerve, characterized by reduced myelin sheath thickness and axon diameter compared with the NAIVE group, indicating damage to both myelin and axonal compartments (Figure 2A,C).

Myelin sheath thickness was markedly reduced after CCI, corresponding to approximately 27.7% of the value observed in NAIVE animals. In the CCI + PBMT group, myelin sheath thickness increased to approximately 50.4% of the NAIVE value, representing an increase of approximately 81.5% relative to untreated CCI animals. Although this finding suggests partial restoration of myelin following PBMT, the difference between the CCI and CCI + PBMT groups was not statistically significant.

A similar trend was observed for axon diameter. CCI reduced axon diameter to approximately 56.0% of the NAIVE value, whereas the CCI + PBMT group reached approximately 80.5% of the control value, corresponding to an increase of approximately 43.7% relative to untreated CCI animals. Despite this apparent improvement, no statistically significant difference was detected between the CCI and CCI + PBMT groups.

Total nerve fiber diameter did not differ significantly among the experimental groups (Figure 2B). Values in the CCI group corresponded to approximately 74.6% of those observed in the NAIVE group, whereas the CCI + PBMT group reached approximately 89.6% of the control value. However, PBMT did not produce a statistically significant effect on this parameter.

Overall, the morphometric findings complement the ultrastructural observations obtained by transmission electron microscopy. CCI induced marked degenerative changes in the sciatic nerve, whereas PBMT was associated with partial preservation of myelin and axonal morphology. Although these improvements were evident morphologically, they were not accompanied by statistically significant recovery of the morphometric parameters evaluated at the time of tissue collection.

### 3.3. Evaluation of Ngf and P0 Immunoreactivity in the Sciatic Nerve

**Figure 3:**
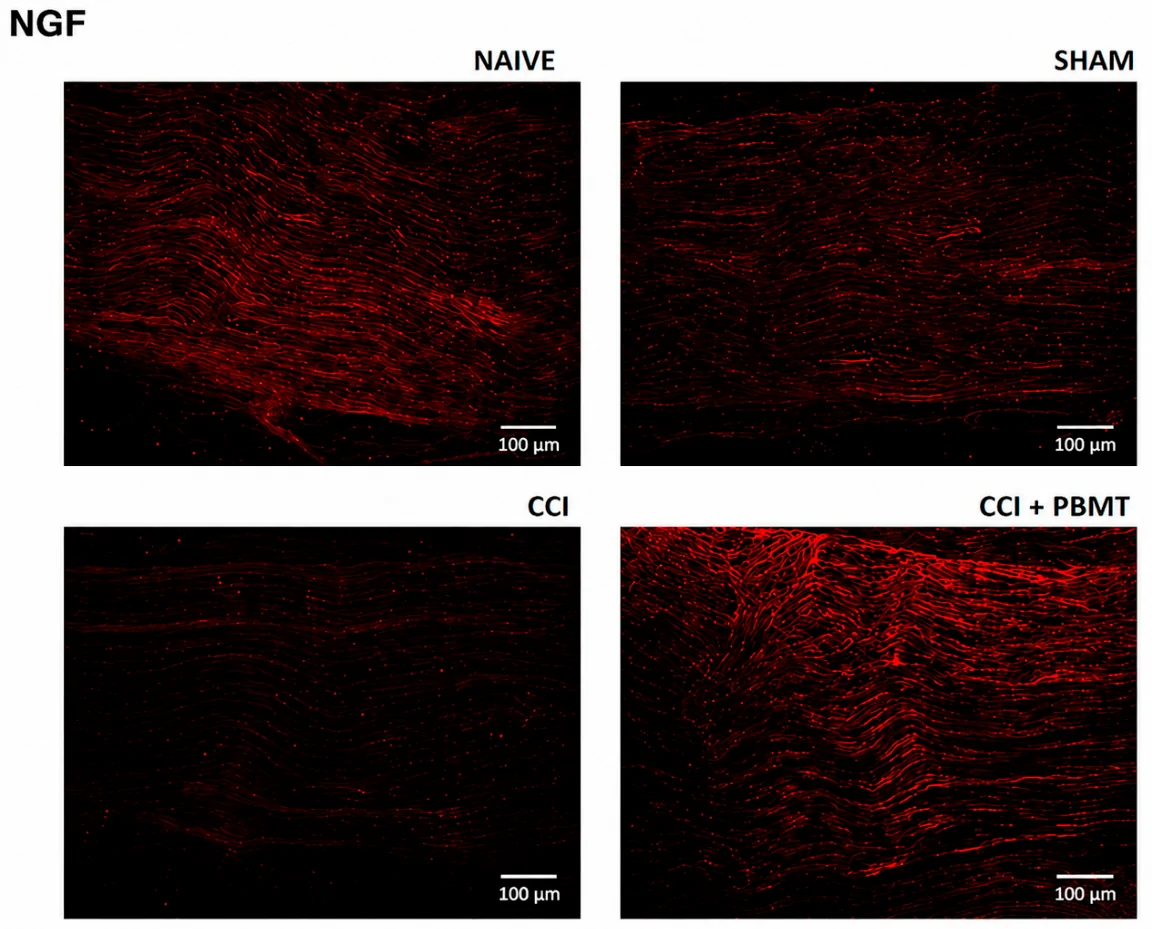
**Effect of photobiomodulation therapy (PBMT) on NGF immunoreactivity in the sciatic nerve following chronic constriction injury (CCI).** Representative immunofluorescence images showing NGF immunoreactivity in the NAIVE, SHAM, CCI, and CCI + PBMT groups. Basal NGF immunoreactivity was observed in the NAIVE and SHAM groups, whereas CCI markedly reduced NGF staining. PBMT increased NGF immunoreactivity compared with untreated CCI animals, suggesting partial restoration of local neurotrophic support following peripheral nerve injury. Images were acquired using a 20× objective. Scale bar = 100 µm.

**Figure 4:**
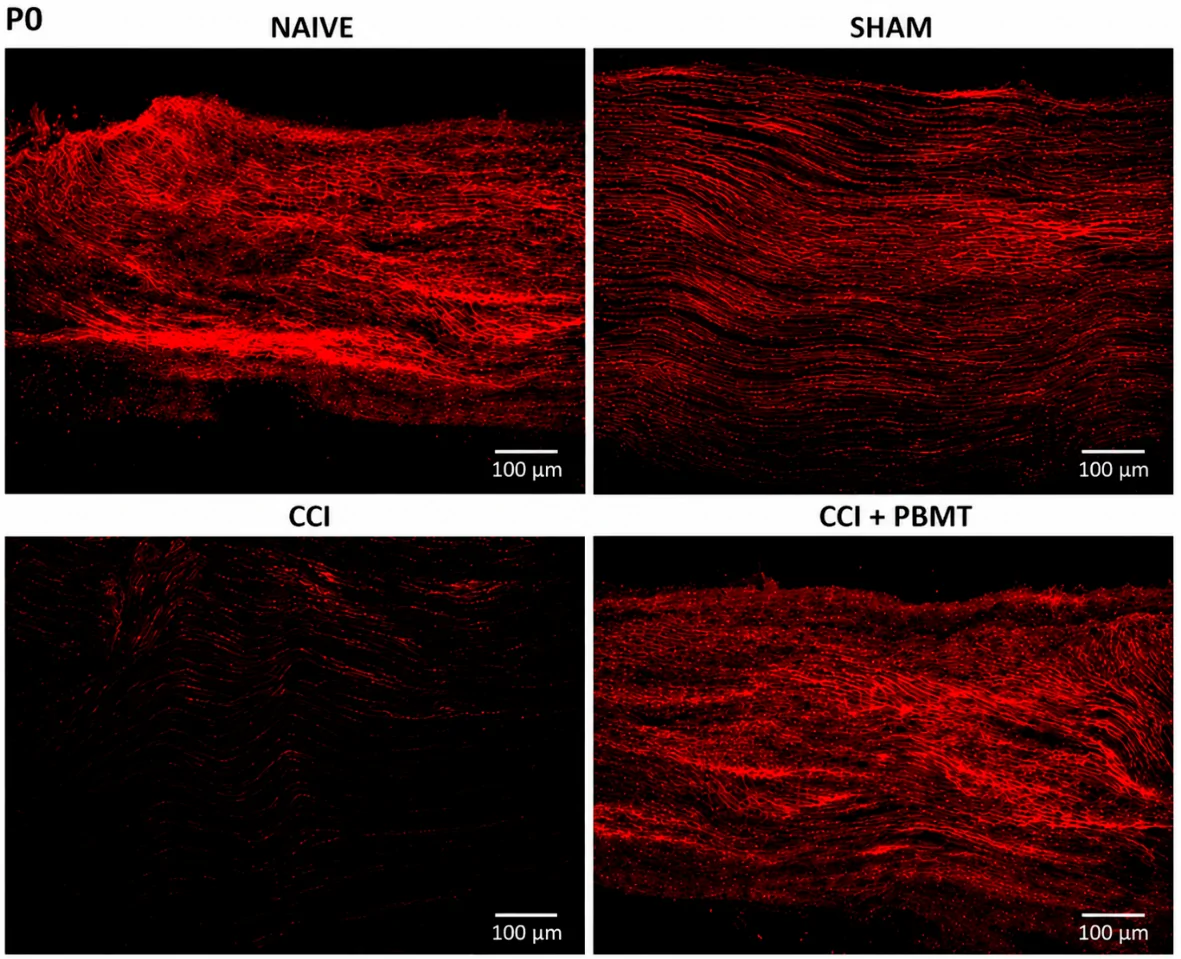
**Effect of photobiomodulation therapy (PBMT) on P0 immunoreactivity in the sciatic nerve following chronic constriction injury (CCI).** Representative immunofluorescence images showing P0 immunoreactivity in the NAIVE, SHAM, CCI, and CCI + PBMT groups. The NAIVE and SHAM groups exhibited staining patterns consistent with preserved myelinated fibers, whereas CCI markedly reduced P0 immunoreactivity. PBMT increased P0 immunoreactivity compared with untreated CCI animals, suggesting preservation and/or restoration of myelin-associated components following peripheral nerve injury. Images were acquired using a 20× objective. Scale bar = 100 µm.

In the control groups (NAIVE and SHAM), NGF and P0 exhibited homogeneous immunoreactivity throughout the sciatic nerve, consistent with preserved nerve architecture and myelinated fibers (Figure 3 and Figure 4). Following chronic constriction injury (CCI), both markers showed reduced immunostaining intensity compared with controls. Reduced NGF immunoreactivity suggests decreased local neurotrophic support after peripheral nerve injury, whereas reduced P0 staining is consistent with disruption of myelin-associated components, corroborating the ultrastructural degenerative changes identified by transmission electron microscopy.

In contrast, animals treated with photobiomodulation therapy (CCI + PBMT) exhibited increased immunoreactivity for both NGF and P0 compared with untreated CCI animals. NGF staining appeared more intense and widely distributed, while P0 immunoreactivity was also enhanced, suggesting preservation and/or restoration of myelin-associated structures. Collectively, these qualitative observations indicate that PBMT may contribute to the preservation of neurotrophic support and peripheral myelin integrity following sciatic nerve injury.

### 3.4. Western Blot Analysis

Western blot analysis was performed to quantitatively evaluate NGF and P0 protein expression in the sciatic nerve following chronic constriction injury (CCI) and photobiomodulation therapy (PBMT).

**Figure 5:**
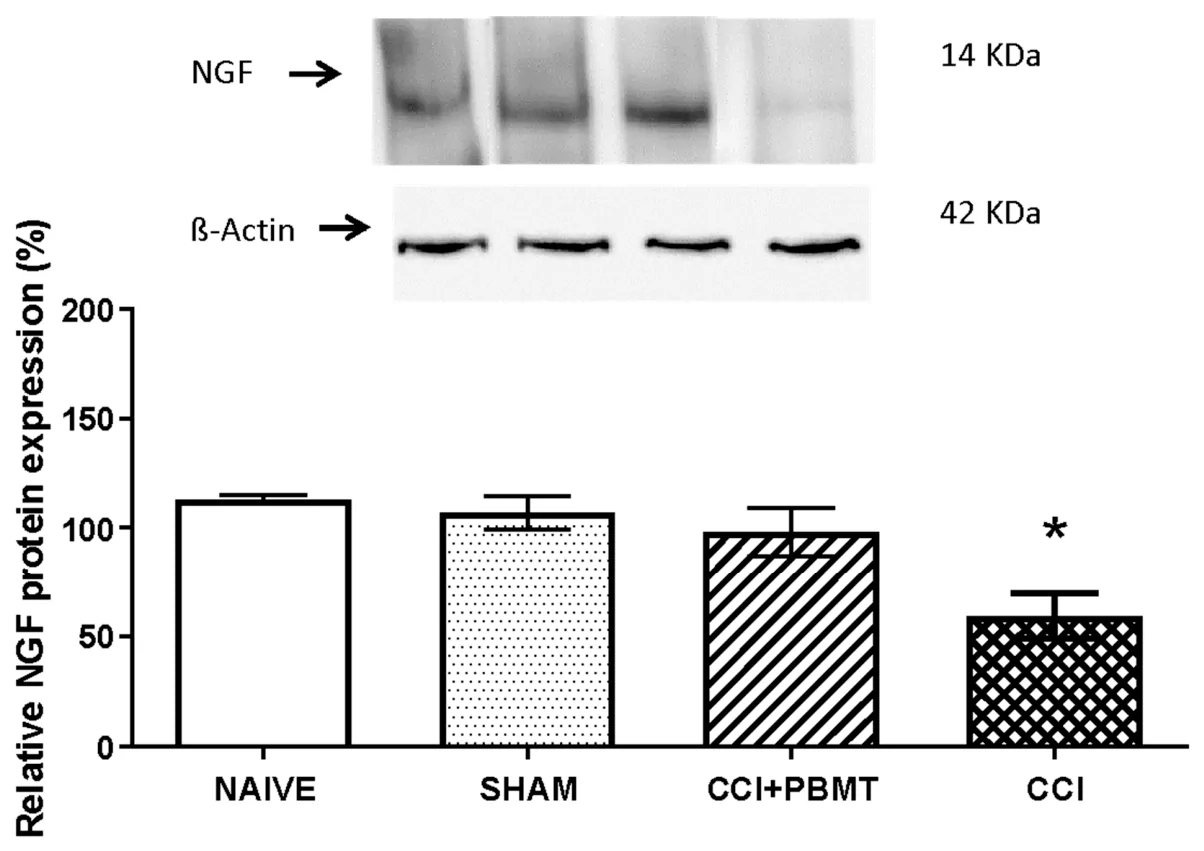
**Effect of photobiomodulation therapy (PBMT) on NGF protein expression in the sciatic nerve following chronic constriction injury (CCI).** Representative Western blots and quantitative densitometric analysis of NGF protein expression normalized to β-actin in the NAIVE, SHAM, CCI, and CCI + PBMT groups. CCI markedly reduced NGF protein expression compared with the control groups, whereas PBMT significantly increased NGF expression, restoring protein levels to values comparable with those of the control groups. Data are presented as mean ± SEM. Statistical analysis was performed using one-way ANOVA followed by Tukey’s post hoc test. * *p* < 0.05 versus NAIVE, SHAM, and CCI + PBMT.

NGF protein expression was markedly reduced following chronic constriction injury. Compared with the NAIVE group (100%), NGF expression in the CCI group decreased to approximately 45% of control levels, representing a reduction of nearly 55% after peripheral nerve injury (Figure 5). In contrast, animals treated with PBMT exhibited a substantial recovery of NGF expression, reaching approximately 96% of the NAIVE value, corresponding to an increase of approximately 113% compared with untreated CCI animals. No significant differences were observed between the NAIVE and SHAM groups, whereas NGF expression in the CCI + PBMT group returned to levels comparable to those of the controls.

**Figure 6:**
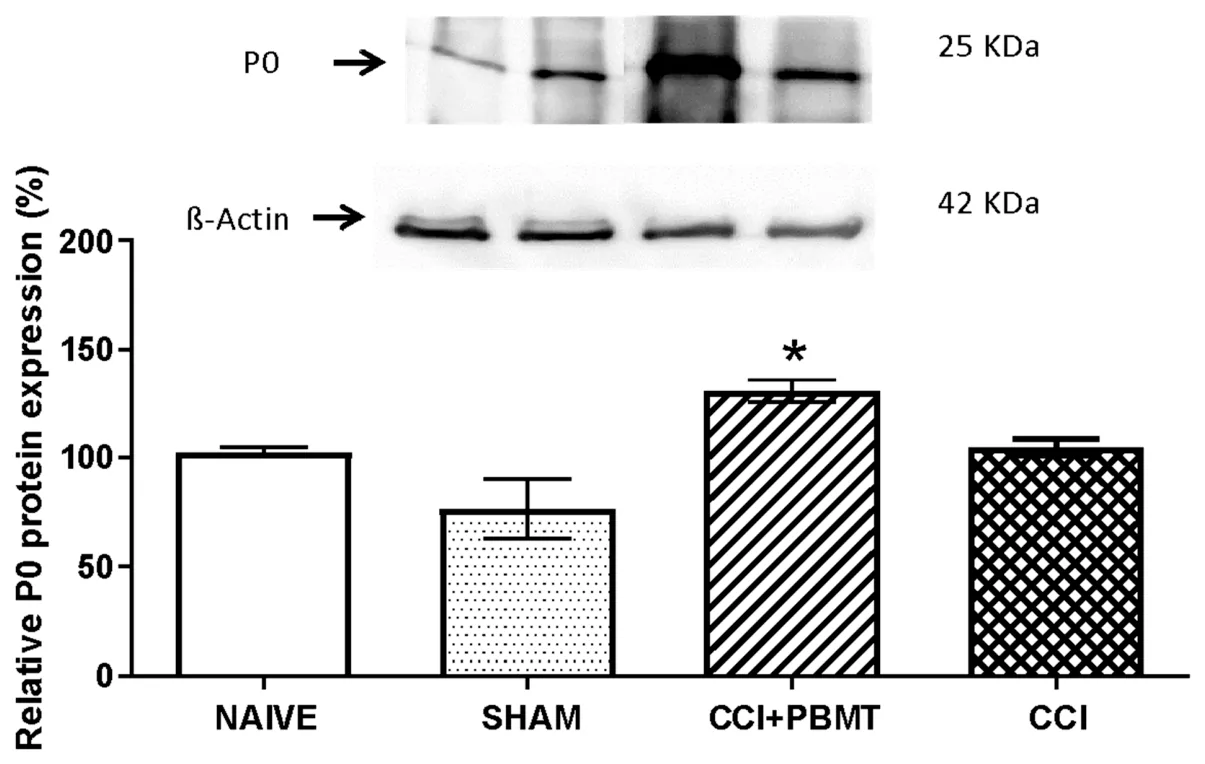
**Photobiomodulation increases P0 protein expression in the sciatic nerve following chronic constriction injury.** Representative Western blots and quantitative densitometric analysis of P0 expression. No significant differences in P0 expression were observed among the NAIVE, SHAM, and CCI groups. However, PBMT significantly increased P0 protein expression compared with all other experimental groups, indicating a beneficial effect of the therapy on proteins associated with peripheral myelin integrity. Data are presented as mean ± SEM. * *p* < 0.05 versus all other experimental groups.

P0 protein expression exhibited a different pattern. Although SHAM and CCI animals showed values corresponding to approximately 70% and 63% of the NAIVE group, respectively, these differences were not statistically significant. In contrast, PBMT increased P0 expression to approximately 113% of control levels, representing an increase of approximately 80% compared with untreated CCI animals. This increase was statistically significant and was consistent with the enhanced P0 immunoreactivity observed by immunofluorescence as well as the ultrastructural evidence of myelin preservation and repair identified by transmission electron microscopy (Figure 6).

Taken together, these findings demonstrate that PBMT restored NGF expression following peripheral nerve injury while simultaneously promoting a significant increase in P0 protein expression, supporting its role in neurotrophic signaling and myelin remodeling during peripheral nerve repair.

## 4. Discussion

The present study investigated the effects of photobiomodulation therapy (PBMT) on the ultrastructural and molecular alterations induced by chronic constriction injury (CCI) of the sciatic nerve. Our findings demonstrate that PBMT restored NGF expression, increased P0 protein levels, and promoted ultrastructural features consistent with axonal regeneration and remyelination. Collectively, these findings suggest that PBMT creates a biological environment favorable for peripheral nerve repair by modulating neurotrophic support and myelin-associated mechanisms.

The antinociceptive effects of PBMT in the CCI model have been previously demonstrated by our group [17]. Therefore, the present study was designed to extend those findings by investigating the structural and molecular mechanisms underlying peripheral nerve repair following laser therapy. The CCI model reproduces several pathological features of human peripheral neuropathies, including inflammation, axonal degeneration, demyelination, and persistent neuronal sensitization [6,11]. Similar beneficial effects of PBMT have also been reported in models of dorsal root ganglion compression, peripheral neuropathy, and inflammatory lesions, where treatment reduced hyperalgesia and improved functional recovery [17,19,27,28,29,30]. The present findings extend these observations by demonstrating that functional improvement is accompanied by molecular and ultrastructural changes indicative of neural repair.

Transmission electron microscopy revealed extensive ultrastructural alterations following CCI, including disorganization of nerve fibers and morphological features characteristic of Wallerian degeneration. In contrast, PBMT-treated animals exhibited greater preservation of nerve architecture and myelinated fibers displaying ultrastructural features consistent with axonal regeneration and remyelination. These observations agree with previous studies demonstrating that photobiomodulation stimulates axonal regeneration, enhances Schwann cell activity, and accelerates peripheral nerve repair after injury [31,32,33,34]. Morphometric analysis confirmed that CCI induced significant reductions in myelin sheath thickness and axonal diameter. Although complete morphometric recovery was not observed, PBMT increased myelin sheath thickness by approximately 82% and axonal diameter by approximately 44% compared with untreated CCI animals, despite the absence of statistical significance. This apparent discrepancy likely reflects different stages of nerve repair, in which molecular and qualitative morphological changes precede complete quantitative restoration of nerve morphology, as previously described during peripheral nerve regeneration [8,22,23,34]. One of the principal findings of the present study was the restoration of NGF expression following PBMT. NGF is a key neurotrophin involved in neuronal survival, axonal growth, phenotypic maintenance, and communication between neurons and Schwann cells. Reduced NGF expression following CCI is consistent with previous studies showing impaired neurotrophic support after peripheral nerve injury [35,36,37]. The restoration of NGF expression after PBMT suggests that laser therapy may enhance the neurotrophic environment required for axonal regeneration and peripheral nerve repair.

PBMT increased P0 protein expression by approximately 80% compared with untreated CCI animals. P0 is the major structural glycoprotein of peripheral myelin responsible for myelin compaction and stability. Because P0 expression depends on Schwann cell differentiation and myelin formation, its upregulation suggests activation of remyelination mechanisms during nerve repair. This interpretation is supported by the increased P0 immunoreactivity and the ultrastructural evidence of preserved myelinated fibers observed after treatment and is consistent with previous studies demonstrating that photobiomodulation enhances Schwann cell activity, promotes myelin remodeling, modulates neuropeptide expression, and regulates mitochondrial and inflammatory pathways involved in peripheral nerve regeneration [13,22,23,34,38,39]. The convergence of ultrastructural, morphometric, immunofluorescence, and Western blot findings substantially strengthens the interpretation of the present study. Although each technique provides distinct but complementary biological information, together they consistently demonstrate that PBMT favors peripheral nerve regeneration by preserving axonal integrity, enhancing neurotrophic support, and stimulating myelin remodeling. Notably, the morphological improvements observed by transmission electron microscopy were accompanied by increased NGF and P0 expression, reinforcing the biological consistency of the regenerative response. The agreement among these independent methodological approaches increases the robustness of the present findings and supports the proposed mechanisms underlying PBMT-induced peripheral nerve repair.

Collectively, the present findings are in agreement with previous experimental studies demonstrating that PBMT promotes peripheral nerve regeneration through multiple biological mechanisms, including modulation of Schwann cell activity, reduction in neuroinflammation, enhancement of axonal regeneration, and stimulation of myelin remodeling. The restoration of NGF together with increased P0 expression observed in the present study extends these previous observations by providing additional molecular evidence supporting the regenerative effects of PBMT.

Although the present findings were obtained in an experimental model, they further support the growing body of evidence indicating that photobiomodulation represents a promising noninvasive therapeutic strategy for peripheral nerve repair. Recent preclinical studies, narrative reviews, and randomized clinical investigations have demonstrated that PBMT may improve nerve regeneration, neurosensory recovery, and functional outcomes following peripheral nerve injuries. Nevertheless, successful clinical translation will require optimization and standardization of irradiation parameters, treatment timing, dosimetry, and standardized treatment protocols before widespread clinical application [40,41,42].

Although the present study was not designed to establish causal relationships, the concomitant restoration of NGF expression, increased P0 levels, and ultrastructural evidence of regeneration support the hypothesis that PBMT promotes peripheral nerve repair through complementary neurotrophic and remyelinating mechanisms. These molecular and structural changes provide a biological basis for the functional recovery previously reported in this experimental model and reinforce the concept that photobiomodulation promotes both neuroprotection and peripheral nerve regeneration.

An additional limitation of the present study is that immunofluorescence was used as a qualitative approach, and quantitative fluorescence intensity analysis was not performed. Nevertheless, the immunofluorescence findings were fully consistent with the Western blot and ultrastructural analyses, providing complementary qualitative evidence consistent with the Western blot findings on NGF restoration and P0 expression. Future studies incorporating quantitative fluorescence analysis may further strengthen the interpretation of these findings.

Some limitations should be acknowledged. The study was performed exclusively in male rats and evaluated a single PBMT protocol, limiting extrapolation to different biological conditions. Furthermore, no functional electrophysiological assessment was performed, preventing direct correlation between structural recovery and nerve conduction. In addition, tissue analysis was restricted to a single time point after treatment, precluding evaluation of long-term nerve remodeling. Future studies should investigate different irradiation parameters, longer follow-up periods, and additional molecular targets involved in axonal regeneration, Schwann cell plasticity, and inflammatory modulation to further clarify the mechanisms underlying PBMT-induced nerve repair.

## 5. Conclusions

Photobiomodulation therapy (PBMT) promoted molecular and ultrastructural changes consistent with peripheral nerve repair following chronic constriction injury of the sciatic nerve. PBMT restored NGF expression, increased P0 protein levels, and induced ultrastructural features indicative of axonal regeneration and remyelination. Although complete morphometric recovery was not achieved during the experimental period, the convergence of morphometric, ultrastructural, immunofluorescence, and Western blot findings indicates that PBMT creates a biological environment that favors peripheral nerve regeneration. Collectively, these findings provide new evidence that the regenerative effects of PBMT involve complementary neurotrophic and myelin-associated mechanisms, reinforcing its therapeutic potential as a noninvasive strategy for the treatment of peripheral nerve injuries and neuropathies.

## Figures and Tables

**Table 1:** **Photobiomodulation therapy (PBMT) parameters.** Technical specifications of the laser device and irradiation protocol used throughout the experimental period, including laser source, wavelength, emission frequency, beam area, energy delivered per irradiation point and per treatment session, energy density, peak power, number of irradiation points, treatment schedule, and total energy administered.

Parameter	Value
Laser device	LASERPULSE Diamond^®^
Laser source	GaAs
Wavelength	904 nm
Emission frequency	9500 Hz
Beam area	0.17 cm^2^
Energy delivered	1.02 J per point/9.18 J per session
Energy density	6 J/cm^2^
Peak power	70 W
Irradiation points	9
Number of sessions	10
Treatment schedule	Alternate days
Total energy delivered	91.9 J

## Data Availability

The data presented in this study are available from the corresponding author upon reasonable request.
